# Modeling and validation of oviposition by a polyphagous insect pest as a function of temperature and host plant species

**DOI:** 10.1371/journal.pone.0274003

**Published:** 2022-09-02

**Authors:** Hyoseok Lee, William M. Wintermantel, John T. Trumble, Trevor M. Fowles, Christian Nansen

**Affiliations:** 1 Department of Entomology and Nematology, University of California, Davis, CA, United States of America; 2 US Department of Agriculture, Agricultural Research Service, Salinas, CA, United States of America; 3 Department of Entomology, University of California, Riverside, CA, United States of America; University of Carthage, TUNISIA

## Abstract

Modeling oviposition as a function of female insect age, temperature, and host plant suitability may provide valuable insight into insect population growth of polyphagous insect pests at a landscape level. In this study, we quantified oviposition by beet leafhoppers, *Circulifer* (= *Neoaliturus*) *tenellus* (Baker) (Hemiptera: Cicadellidae), on four common non-agricultural host plant species [*Erodium cicutarium* (L.) L’Hér. (Geraniaceae), *Kochia scoparia* (L.) Schrader (Amaranthaceae), *Plantago ovata* Forsskál (Plantaginaceae), and *Salsola tragus* L. (Amaranthaceae)] at two constant temperature conditions. Additionally, temperature-based oviposition models for each host plant species were validated, under semi-field and greenhouse conditions. We found that *K*. *scoparia* was the most suitable host plant, and optimal temperature for oviposition was estimated to be 30.6°C. Accordingly, beet leafhoppers appear to be well-adapted to high-temperature conditions, so increasing temperatures due to climate change may favor population growth in non-agricultural areas. Maximum total fecundity (*R*_*m*_) was used as an indicator of relative suitability of host plants. *S*. *tragus* has been considered an important non-agricultural host plant, however, we found that *S*. *tragus* and *E*. *cicutarium* have lower *R*_*m*_ compared to *K*. *scoparia* and *P*. *ovata*. The combination of detailed experimental oviposition bioassays, modeling, and model validation is considered widely relevant and applicable to host plant assessments and modeling of population dynamics of other polyphagous insect pests.

## Introduction

Polyphagy is a significant challenge regarding development of effective management strategies for economically important insect pests of agricultural crops [1], as it may require coordination of management efforts across multiple crops and possibly in both agricultural and non-agricultural habitats [2]. For polyphagous insect pests, it is of paramount importance to characterize and ideally quantify relative importance and potential of different host plant species [3]. Stated boldly, if an insect pest is known to be able to successfully complete its life cycle on multiple host plant species, are they contributing equally to the overall insect pest population dynamics? This is one of the basic questions addressed in this study, and a reasonable initial approach is to compare oviposition by individual female insects on multiple host plant species. For example, oviposition by diamondback moths, *Plutella xylostella* (L.) (Lepidoptera: Plutellidae) was tested on six different Brassicaceae (order Brassicales) crops including cabbage (*Brassica oleracea* L. var. *capitata*), cauliflower (*Brassica oleracea* L. var. *botrytis*), radish (*Raphanus sativus* L.), turnip (*Brassica rapa* L.), mustard (*Brassica compestris* L.), and canola (*Brassica napus* L. var. *canola*) [4]. The authors concluded that canola was the most suitable host plant for *P*. *xylostella* population increase due to higher total fecundity as well as a higher percentage of survival. In addition, life-table data for tobacco cutworm, *Spodoptera litura* (Fabricius) (Lepidoptera: Noctuidae), were collected on multiple green manure cover crops including sesbania (*Sesbania roxburghii* Merr.) (Fabales: Fabaceae), sunn hemp (*Crotalaria juncea* L.) (Fabales: Fabaceae), and rapeseed (*Brassicae campestris* L.) (Brassicales: Brassicaceae) [5]. Tobacco cutworms laid about 1.5 times more eggs on sesbania than other green manure cover crops. The authors emphasized the importance of sesbania fields as major sources of tobacco cutworm and need of an area-wide pest management program based on host suitability. Additionally, assessments of relative suitability of host plant species can be used to characterize and model insect pest population dynamics as a function of vegetation composition.

In addition to suitability of host plant species, oviposition by insects is influenced by age and temperature, and the relationship of these variables with oviposition is typically asymmetric and unimodal [6–8]. Accordingly, insect oviposition under fluctuating temperature conditions has been described by four components: temperature-dependent total fecundity, temperature-dependent female aging rate, age-specific cumulative oviposition rate, and age-specific survival of female adults [9–11]. Total fecundity refers to the total number of eggs laid by individual females in their entire adult lifespan. Oviposition rate and survival of female adults are mainly dependent on their age [12], and their aging rate is affected by temperature [7]. Taylor [11] measured insect age by using the physiological time concept, accumulation of temperature-dependent development (aging) rate at each instant in time. Cumulative oviposition rate refers to the cumulative proportion of number of eggs laid by individual females at an age to its total fecundity. Survival of female adults refers to survival probability of individual females at an age. Age-specific cumulative oviposition rate and survival have not been investigated in many insects, but such data are needed to describe insect oviposition under fluctuating temperature conditions. To accurately describe relationships between temperature and total fecundity, non-linear functions such as Lactin [13], extreme value [10, 14], and Gaussian [15] functions have been used. Age-specific cumulative oviposition rate and survival have been modeled using Gompertz [16], sigmoid [15], or Weibull [17] functions.

In this study, beet leafhoppers, *Circulifer* (= *Neoaliturus*) *tenellus* (Baker) (Hemiptera: Cicadellidae) were used as a model insect. Beet leafhoppers have a wide host range, including non-agricultural trees, shrubs, and annual plant species [18, 19]. Beet leafhopper is an economically important insect pest as it vectors beet curly top virus (BCTV), which may cause significant yield losses in economically important crops including tomato (*Solanum lycopersicum* L.) (Solanales: Solanaceae), sugar beet (*Beta vulgaris* L.) (Caryophyllales: Amaranthaceae), pepper (*Capsicum annuum* L.) (Solanales: Solanaceae), spinach (*Spinacia oleracea* L.) (Caryophyllales: Amaranthaceae), and common bean (*Phaseolus vulgaris* L.) (Fabales: Fabaceae) [20]. In North America, beet leafhoppers are the only known vectors of BCTV [21], and they transmit BCTV to crops from non-agricultural host plants in spring, after migrating from non-agricultural habitats into agricultural landscapes [22]. In addition, multiple phytoplasmas associated with serious plant diseases are transmitted by beet leafhoppers in North America and Palearctic regions [23, 24]. Thus, evaluation of suitability of non-agricultural host plants for oviposition of beet leafhopper is needed to effectively characterize and model beet leafhopper population growth and ultimately, to develop sustainable management strategies for this important insect pest. In the current study, we quantified oviposition by beet leafhoppers on four non-agricultural host plant species, *Erodium cicutarium* (L.) L’Hér. (Geraniales: Geraniaceae), *Kochia scoparia* (L.) Schrader (Caryophyllales: Amaranthaceae), *Plantago ovata* Forsskál (Lamiales: Plantaginaceae), and *Salsola tragus* L. (Caryophyllales: Amaranthaceae). These host plants were selected because beet leafhoppers commonly use *E*. *cicutarium*, *P*. *ovata*, and *S*. *tragus* as winter and spring non-agricultural host plants and *K*. *scoparia* as a summer non-agricultural host plant [18]. Oviposition models (i.e., temperature-dependent total fecundity, temperature-dependent female aging rate, age-specific cumulative oviposition rate, and age-specific survival) were constructed for each host plant species. In addition, these models were validated under fluctuating temperature conditions (semi-field and greenhouse conditions), and the relative importance of the non-agricultural host plant species in terms of beet leafhopper management was discussed.

## Materials and methods

### Oviposition and longevity experiments

**Fig 1:**
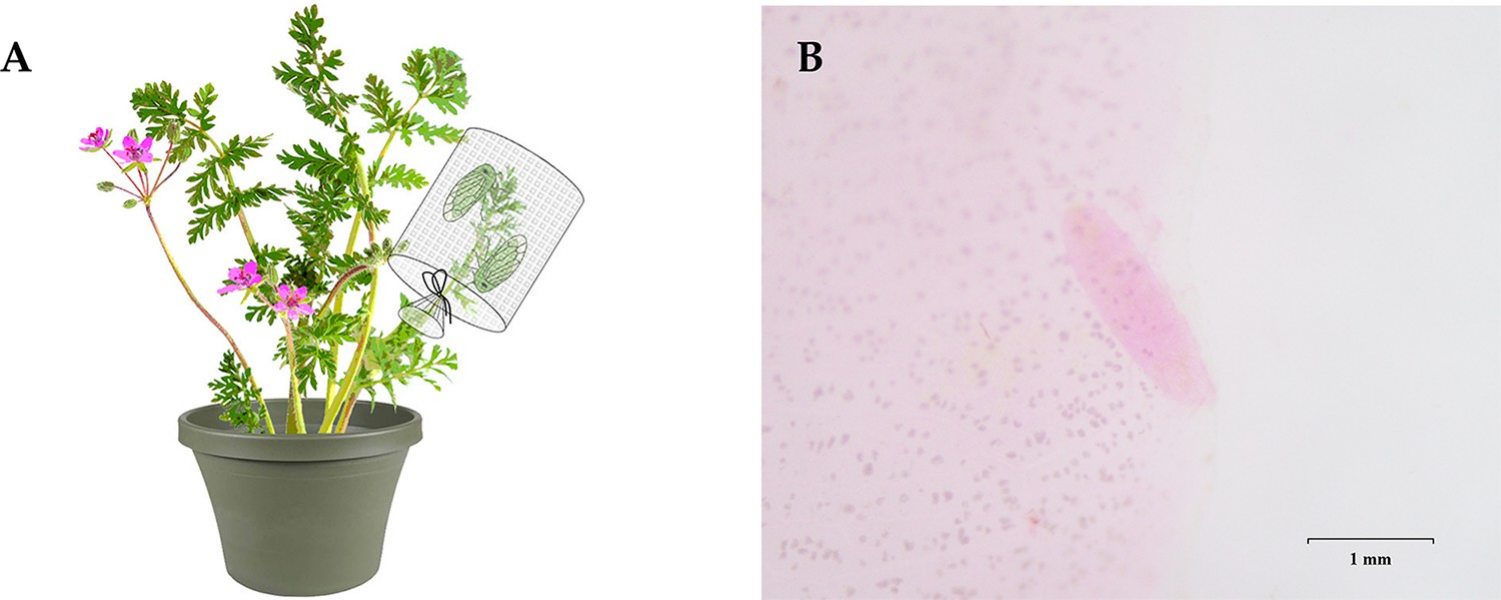
Diagrammatic illustration of the experimental design (A). A beet leafhopper egg laid in a sugar beet leaf stained with McBryde’s solution (B).

Beet leafhopper colonies were reared on sugar beet (*Beta vulgaris* L.) (Caryophyllales: Amaranthaceae). Four non-agricultural host plants, *E*. *cicutarium*, *K*. *scoparia*, *P*. *ovata*, and *S*. *tragus*, were grown in pots (d = 11 cm, h = 9.5 cm) under greenhouse conditions (25 ± 5°C, 50 ± 10 RH). Individual pairs of newly emerged beet leafhopper adults (< 24 h-old) were transferred into meshed cages (d = 6 cm, h = 10 cm) on a tip of a branch of each non-agricultural host plant (Fig 1A). In total, 110 meshed cages were maintained at two constant temperatures (30 or 35°C) in growth chambers. Beet leafhopper males that died during early stages of the experiment (< 1 week) were replaced with new males to ensure mating. Host plant leaves were collected weekly, and meshed cages were transferred to new tips until females died. Counting of eggs laid was facilitated by staining of leaves using a modified McBryde’s solution [25], consisting of 0.2% (wt/vol) acid fuchsin in a mixture of 95% ethanol and glacial acetic acid (1:1 vol/vol) for 24 hours [26]. Leaves were cleared in a clearing solution consisting of distilled water, 99% glycerol, and 95% lactic acid (1:1:1 vol/vol/vol) at 95°C for four hours. Stained eggs were counted under a binocular stereomicroscope (Olympus SZ51; Olympus, Tokyo, Japan) (Fig 1B). Cumulative oviposition rate and survival of female adults were recorded every week for each combination of host plant and temperature condition (n = 11 to 15 cages/temperature/plant). We performed statistical analyses using R version 4.1.2 [27]. A Generalized Linear Mixed-Effects Model (GLMM) was used with a negative binomial distribution with host plant species, temperature, and their interaction as fixed effects. In addition, a random effect of the date on which the replicates had been conducted was incorporated into the model using ‘glmer.nb’ function in the ‘lme4” package [28]. The total fecundity and longevity were the response variables for the analyses. We used Tukey’s HSD test to perform post hoc tests at the *α* = 0.05 level using the ‘emmeans’ package [29]. Bonferroni’s correction was used to obtain adjusted *p*-values.

### Oviposition model components

#### Temperature-dependent total fecundity

Relationship between total fecundity and temperature was described by a Gaussian function [11]:

$$f(T)=R_{m}e^{\left[-\frac{1}{2}({\left(T-T_{max})/k\right)}^{2}\right]}$$ (1)

where *f(T)* is the total number of eggs laid per female at temperature *T* (°C), *R*_*m*_ is the maximum total fecundity, *T*_*max*_ is the temperature (°C) at which the maximum fecundity occurs, and *k* is an estimated parameter defining curve steepness. Total fecundity of beet leafhoppers reared on sugar beet was obtained from Harries and Douglass (1948) [30] and fitted to Eq (1) to estimate parameters. Regarding non-agricultural host plant species, only *R*_*m*_ was re-estimated with the same *T*_*max*_ and *k* parameters estimated as for sugar beet. *R*_*m*_ values for non-agricultural host plant species were compared by performing ANOVA as described above.

#### Temperature-dependent female aging rate

Temperature-dependent female aging rate was modeled based on reciprocals of longevity of female adults reared on sugar beet and non-agricultural host plant species. The following equation was used to describe female aging rate as a function of temperature [31]:

$$r(T)=\sigma *e^{\left(a+bT^{2.5}+cT^{3}\right)}$$ (2)

where *r(T)* is the female aging rate (1/days) at temperature *T* (°C), *σ* is a host-specific parameter indicating a relative influence of non-agricultural host plant species compared to sugar beet (i.e., 1 for sugar beet), and *a*, *b*, and *c* are estimated parameters. Data for modeling female aging rate on sugar beet was obtained from Harries and Douglass (1948) [30] and used to estimate *a*, *b*, and *c* parameters. Subsequently, *σ* was estimated for each non-agricultural host plant species with the same *a*, *b*, and *c* parameters estimated as for sugar beet:

Female age was normalized by accumulating female aging rate as the following equation [9].

$$Px=\sum_{i=0}^{n}r(T_{i})$$ (3)

where *Px* is normalized female age, *n* is days from emergence, *r(T*_*i*_*)* is the female aging rate at temperature *T* (°C) of *i*th day after emergence.

#### Age-specific cumulative oviposition rate

Age-specific cumulative oviposition rate of beet leafhopper was modeled using the Weibull function [15]:

$$p(Px)=1-e^{{-(Px/\alpha )}^{\beta }}$$ (4)

where *p(Px)* is the cumulative rate of eggs laid at normalized female age *Px*, and *α* and *β* are estimated parameters. As female age was normalized, oviposition data obtained under all temperature and non-agricultural host plant conditions were combined and modeled together.

#### Age-specific survival of female adults

Age-specific survival of adult beet leafhopper was modeled using a sigmoid function [15]:

$$s(Px)=\frac{1}{1+e^{(\gamma -Px)/\delta }}$$ (5)

where *s(Px)* is the proportion of live females at normalized female age *Px*, *γ* is the normalized female age at 50% survival, and *δ* is an estimated parameter. Survival data under all temperature and non-agricultural host plant conditions were combined and modeled together.

### Model validation

#### Semi-field and greenhouse data collection

We measured oviposition of beet leafhoppers reared on non-agricultural host plant species under fluctuating temperature conditions. Individual pairs of newly emerged beet leafhoppers (< 24 h-old) were released into meshed cages (d = 6 cm, h = 10 cm) containing non-agricultural host plants (two replicates for each host plant species under different temperature conditions). For *E*. *cicutarium*, *P*. *ovata*, and *S*. *tragus*, meshed cages were maintained under experimental field settings at the University of California, Davis during winter months. Meshed cages for *K*. *scoparia* were maintained in a greenhouse to mimic summer temperature conditions (S1 Table). Host plant leaves were collected weekly for a total of 10 to 15 weeks (depending on longevity of beet leafhopper females), and leaves were stained as described above to count numbers of eggs laid. Daily ambient temperatures were recorded using Hobo loggers (Onset Computer, Co., Bourne, MA, USA) and used to predict beet leafhopper oviposition.

#### Simulation and validation

Daily oviposition at *i*th day on each non-agricultural host plant was considered the product of temperature-dependent total fecundity [*f(T*_*i*_*)*], change in age-specific cumulative oviposition [*p(Px*_*i+1*_*)*–*p(Px*_*i*_*)*], and survival *s*(*Px*). Daily oviposition of beet leafhoppers on sugar beet was simulated at a constant temperature ranging from 10 to 50°C. In addition, weekly oviposition at *n*th week [*F(n)*] was simulated by adding up daily oviposition according to the following equation:

$$F(n)=\sum_{i=n}^{n+6}f(T_{i})[p({Px}_{i+1})-p({Px}_{i})]$$ (6)

Oviposition model outputs for non-agricultural host plant species were compared with the semi-field and greenhouse observation data to validate the models.

## Results

### Total fecundity and longevity on non-agricultural host plants

Both host plant species and temperature significantly affected the total fecundity (host plant species: *χ*^2^ = 811.40, *df* = 3., *p* < 0.001; temperature: *χ*^2^ = 43.32, *df* = 1, *p* < 0.001) (Table 1). However, there was no significant effect of their interaction (*χ*^2^ = 1.38, *df* = 3, *p* = 0.71). Beet leafhoppers laid most eggs when reared on *K*. *scoparia* at both temperatures. Longevity was also significantly influenced by temperature, but there was no significant effect of host plant species and their interaction (host plant species: *χ*^2^ = 6.89, *df* = 3, *p* = 0.08; temperature: *χ*^2^ = 14.53, *df* = 1, *p* < 0.001; their interaction: *χ*^2^ = 1.70, *df* = 3, *p* = 0.64) with beet leafhoppers living significantly longer when reared on *K*. *scoparia* under both temperature conditions.

**Table 1:** Total fecundity (mean ± SD) and longevity (mean ± SD) of beet leafhoppers on four common non-agricultural host plants at constant temperatures.

Plant species	Total fecundity (eggs/female)		Longevity (weeks)	
	30°C	35°C	30°C	35°C
*Erodium cicutarium*	27.15 ± 10.38bA (13)	17.33 ± 4.92bB (15)	5.31 ± 1.38A (13)	3.20 ± 0.77B (15)
*Kochia scoparia*	137.57 ± 20.80aA (14)	102.17 ± 39.49aB (12)	6.43 ± 1.09A (14)	5.00 ± 1.60A (12)
*Plantago ovata*	106.60 ± 13.61aA (15)	79.27 ± 23.86aB (15)	5.53 ± 0.92A (15)	4.27 ± 0.80A (15)
*Salsola tragus*	30.07 ± 5.56bA (15)	20.64 ± 7.83bB (11)	5.13 ± 0.92A (15)	3.82 ± 0.98A (11)

Different lowercase letters (a-b) indicate significant differences in total fecundity of beet leafhoppers on different plant species under the same temperature. There was no significant difference in longevity on different plant species under the same temperature. Different capital letters (A-B) indicate significant differences in total fecundity or longevity of beet leafhoppers at different temperatures within the same plant species. Values in parentheses are sample sizes.

### Oviposition model components

#### Temperature-dependent total fecundity and female aging rate models

**Fig 2:**
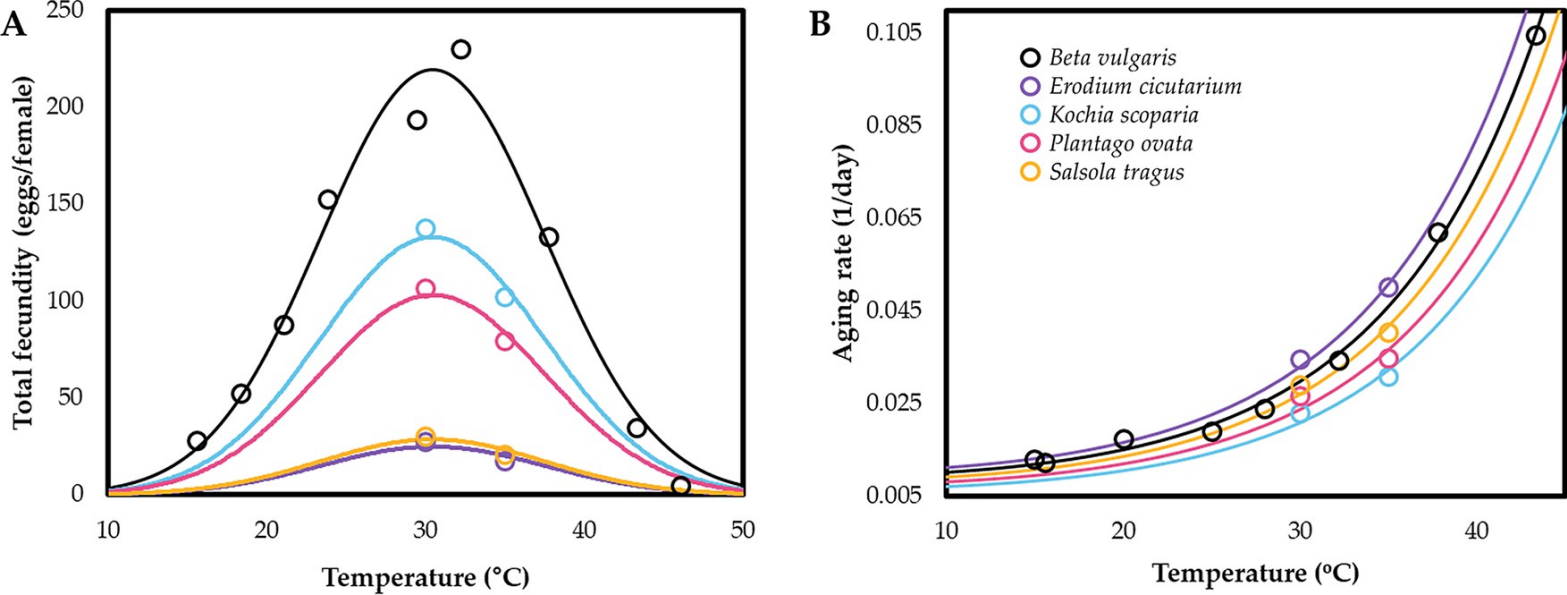
Temperature-dependent (A) total fecundity and (B) aging rate (1/mean longevity) models of female beet leafhoppers on five host plant species. The data points for sugar beet were obtained from the published paper, Harries and Douglass (1948) [30].

Temperature-dependent total fecundity of beet leafhoppers reared on sugar beet was well described by the Gaussian function (*F*[2,8] = 116.0, *p* < 0.001, *adj-r*^*2*^ = 0.966) (Fig 2A, Table 2). Temperature (*T*_*max*_) with the maximum total fecundity was estimated to be 30.6°C. Total fecundity on the non-agricultural host plant species was also well-described by the Gaussian function (1) with the same *T*_*max*_ and various *k* values (Table 2). *K*. *scoparia* showed the highest *R*_*m*_ value among the non-agricultural host plants (*F*[4,7] = 609.0, *p* < 0.001).

**Table 2:** Estimated parameters in total fecundity models of female beet leafhoppers on five host plant species.

Host plant	Parameter	Estimate (SE)	Adjusted r^2^
Sugar beet	*R*_*m*_	216.982 (9.876)	0.966
	*T*_*max*_	30.558 (0.397)	
	*K*	7.182 (0.362)	
*Erodium cicutarium*	*R*_*m*_	24.872 (2.871)	0.717
*Kochia scoparia*	*R*_*m*_	133.050 (5.895)	0.908
*Plantago ovata*	*R*_*m*_	103.146 (4.507)	0.910
*Salsola tragus*	*R*_*m*_	28.610 (2.603)	0.777

The female aging rate model effectively described aging rates (1/longevity) of beet leafhoppers reared on sugar beet (*F*[2,7] = 1010.4, *p* < 0.001, *adj-r*^*2*^ = 0.997) (Fig 2B, Table 3). Regarding female aging rate models for the non-agricultural host plant species, we estimated only *σ* for each non-agricultural host plant species (Table 3). Female aging rates were positively related to temperature for all host plant species (Fig 2B). Only female beet leafhoppers reared on *E*. *cicutarium* showed a higher aging rate than those reared on sugar beet.

**Table 3:** Estimated parameters in female aging rate models of beet leafhoppers on five host plant species.

Host plant	Parameter	Estimate (SE)	Adjusted r^2^
Sugar beet	a	-4.700 (0.121)	0.997
	b	4.506e-04 (1.016e-04)	
	c	-3.837e-05 (1.412e-05)	
*Erodium cicutarium*	*σ*	1.108 (0.032)	0.975
*Kochia scoparia*	*σ*	0.700 (0.046)	0.795
*Plantago ovata*	*σ*	0.796 (0.062)	0.661
*Salsola tragus*	*σ*	0.904 (0.044)	0.912

#### Age-specific cumulative oviposition rate and survival

**Fig 3:**
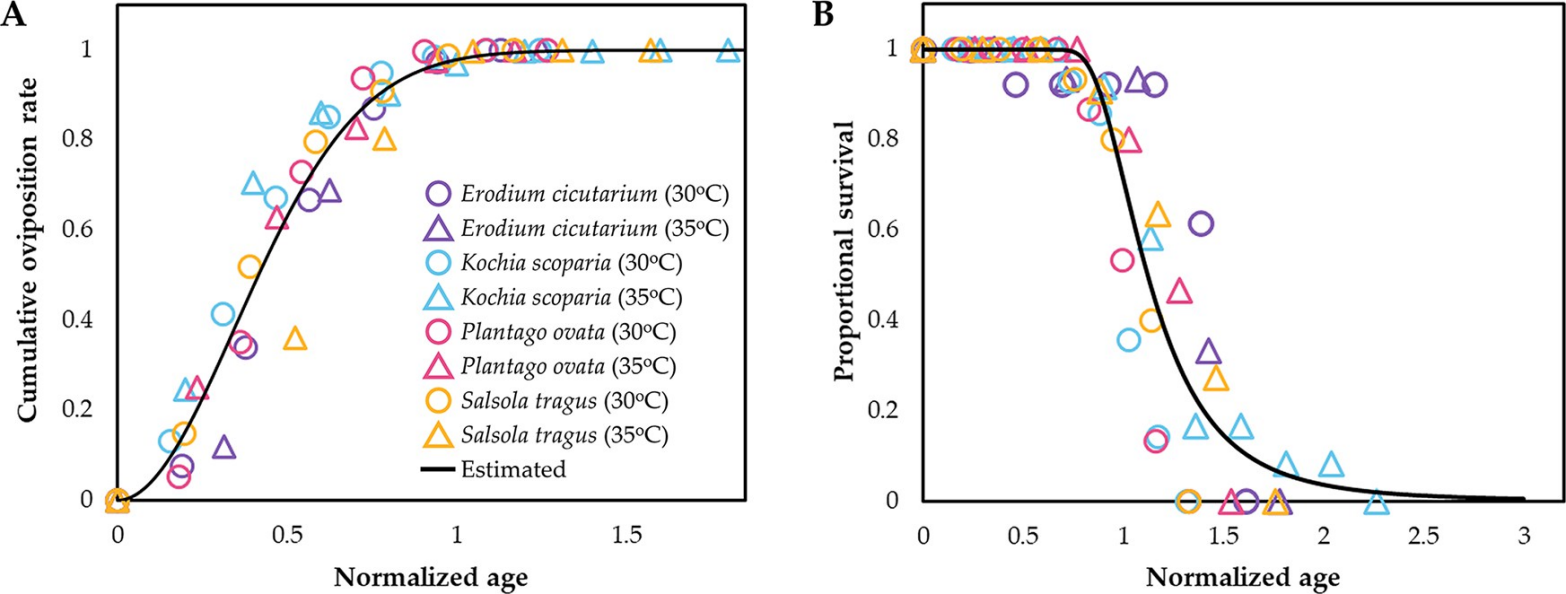
Age-specific (A) cumulative oviposition rate and (B) survival models of female beet leafhoppers on four non-agricultural host plant species at two temperature conditions.

Relationship between cumulative oviposition rate and normalized female age was described well by the Weibull function (*F*[1,57] = 1571.5, *p* < 0.001, *adj-r*^*2*^ = 0.965) (Fig 3A, Table 4). Most eggs were laid before beet leafhoppers reached the normalized female age of 1. Age-specific survival of beet leafhoppers was also described well by the Weibull function (*F*[1,65] = 438.0, *p* < 0.001, *adj-r*^*2*^ = 0.871) (Fig 3B, Table 4). Approximately half of female beet leafhoppers died at the normalized female age of 1.

**Table 4:** Estimated parameters in age-specific cumulative oviposition rate and survival models.

Model	Parameter	Estimate (SE)	Adjusted r^2^
Age-specific cumulative oviposition rate	*α*	0.501 (0.012)	0.965
	*β*	1.957 (0.137)	
Age-specific survival	*γ*	1.042 (0.022)	0.871
	δ	-5.083 (0.696)	

### Simulation and validation

**Fig 4:**
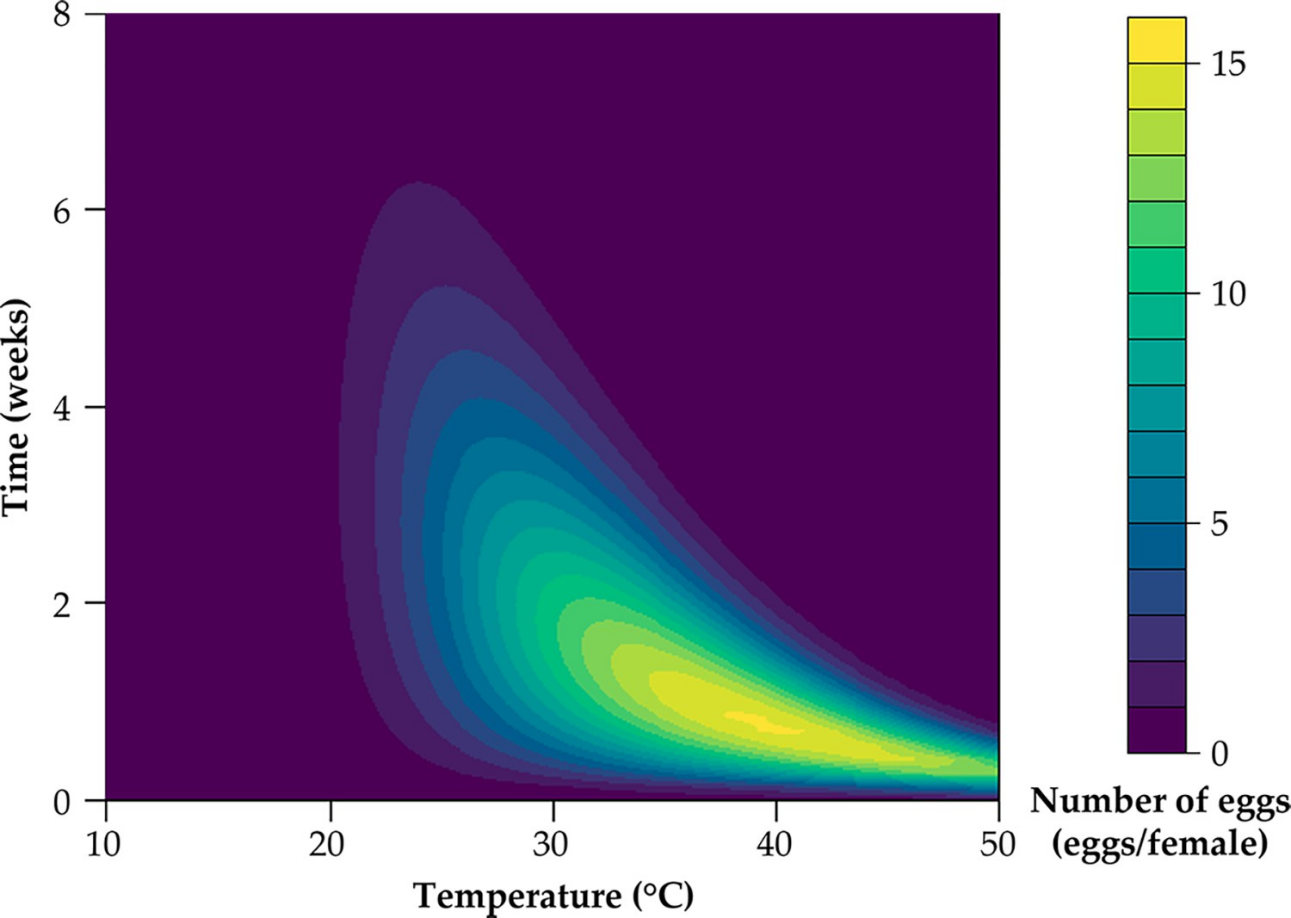
Estimated oviposition of beet leafhoppers on sugar beet in relation to temperature.

**Fig 5:**
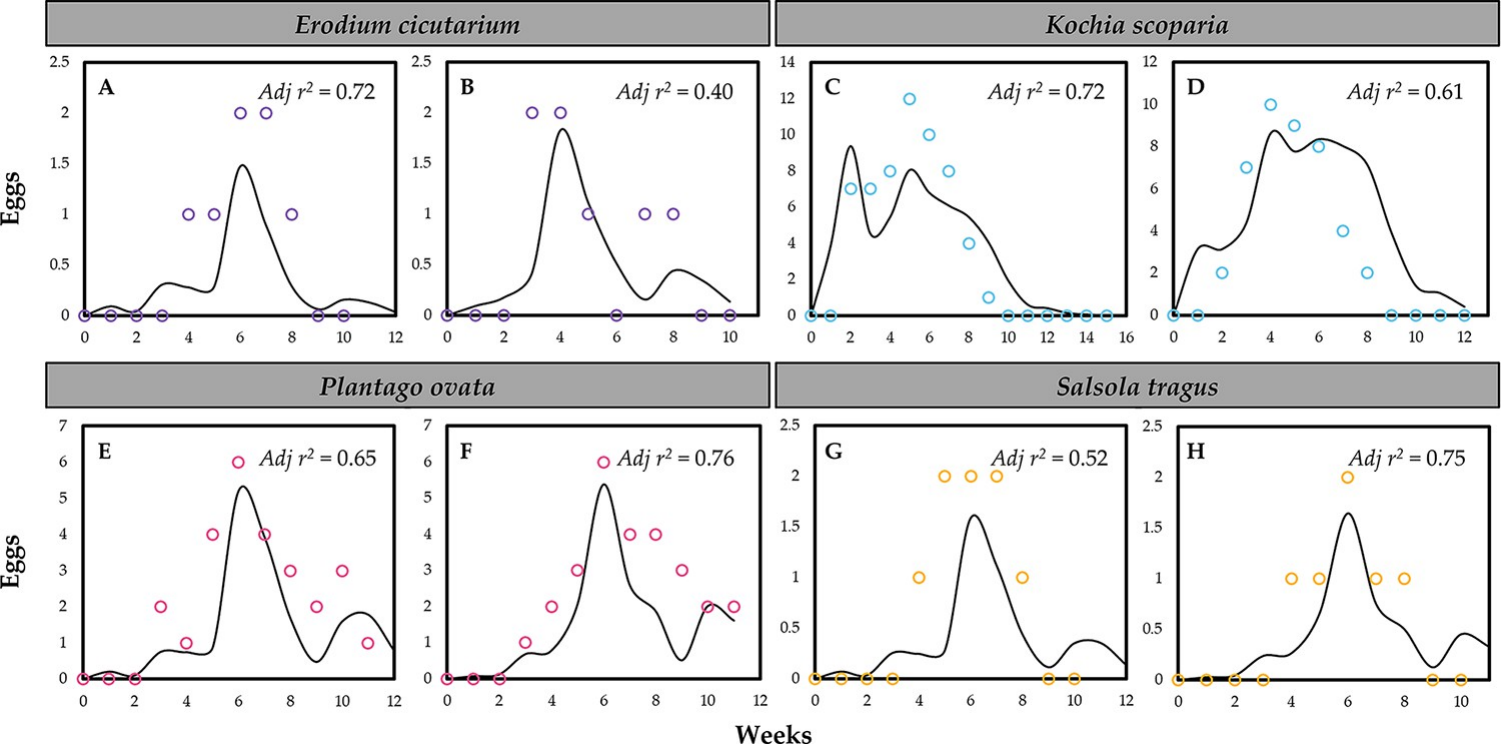
Model validation for oviposition of beet leafhoppers on four non-agricultural host plant species, (A and B) *Erodium cicutarium*, (C and D) *Kochia scoparia*, (E and F) *Plantago ovata*, and (G and H) *Salsola tragus*, under fluctuating temperature conditions (see S1 Table). Open dots and solid lines represent observed oviposition and the oviposition model outputs, respectively.

From simulations, oviposition period increased with decreasing temperature, but maximum weekly oviposition occurred at about 40°C (Fig 4). Oviposition on non-agricultural host plant species under semi-field and greenhouse conditions was compared with the oviposition model outputs (Fig 5 and S2 Table). Predicted oviposition followed the semi-field and greenhouse observation data well, although oviposition models tended to underestimate oviposition compared to the semi-field and greenhouse observation data.

## Discussion

Temperature effects on total fecundity and female aging rate of beet leafhoppers have been assessed only on sugar beet [21], and the results were converted into predictive models to aid in beet leafhopper management [20]. However, the most damaging beet leafhopper populations that spread BCTV to crops are believed to originate from non-agricultural areas adjacent to crop fields [22]. In this study, we described oviposition of beet leafhoppers on four common non-agricultural host plant species and constructed oviposition models which were validated under semi-field and greenhouse conditions. In addition, we offered an important parameter, the maximum total fecundity (*R*_*m*_), for each non-agricultural host plant species, which represents the host suitability for beet leafhopper oviposition. *S*. *tragus* has been considered one of the most important non-agricultural host plant species for population growth of beet leafhopper [32]. However, we found that *S*. *tragus* and *E*. *cicutarium* have a lower potential for beet leafhopper population growth compared to *K*. *scoparia* and *P*. *ovata*. In addition, *Salsola* species are considered less suitable as a reservoir of some BCTV strains [32–34]. Therefore, *K*. *scoparia* and *P*. *ovata* may be more important host plant species to beet leafhopper population dynamics in non-agricultural areas.

In this study, a single host plant species was offered to the beet leafhopper pairs, although plant communities in the real world possess varying degrees of diversity. However, polyphagous insects may exhibit different oviposition behavior depending on plant communities they encounter and their host preference. For example, Romero et al. [35] found out that aster leafhoppers (*Macrosteles quadrilineatus* Forbes) (Hemiptera: Cicadellidae) showed different oviposition behavior when offered multiple host plant species compared to when only one host plant species was given. In a no-choice experiment, no eggs were oviposited on canola by aster leafhoppers [36], but a similar number of eggs was observed on canola as on other more suitable host plant species in a choice experiment. In addition, beet leafhoppers exhibit host plant preference [37] that may affect oviposition behavior depending on vegetation composition. Therefore, future research comparing oviposition behavior of beet leafhoppers with different vegetation compositions may provide insights to enhance our understanding of the potential population increase under diverse plant communities.

We found that total fecundity of beet leafhoppers was positively correlated to temperature until the optimal temperature (30.6°C). Because beet leafhoppers are well-adapted to high-temperature conditions, the temperature for optimal fecundity is higher compared to other insect species [10, 14, 31]. In addition, *K*. *scoparia* and *P*. *ovata* are well adapted to high temperature conditions [38, 39]. Therefore, increasing temperatures due to climate change is likely to favor population growth in non-agricultural areas. It is necessary to estimate the pest status of beet leafhopper in connection with changing environmental conditions and corresponding responses of host plants.

Spatio-temporal modeling of population dynamics as a function of abiotic conditions and vegetation composition will be needed in order to develop and implement precision-guided and sustainable management practices of beet leafhoppers. Furthermore, such modeling efforts will likely require inclusion of beet leafhopper population dynamics in non-agricultural areas. As an example, the California Department of Food and Agriculture manages the Curly Top Virus Control program, which includes extensive and continuous sweep net sampling and airplane-based sprays with malathion in non-agricultural areas in the coastal and central foothills of California. Due to the large geographical scale, region-wide decision support tools are needed to precision-guide both sweep netting efforts and targeted sprays of malathion. Models presented in this study can be integrated into geographical information system (GIS) tools to predict and visualize spatio-temporal dynamics of beet leafhopper densities in non-agricultural areas so that emerging beet leafhopper hotspots can be detected.

## Conclusions

Both temperature and host plant species significantly affected oviposition of beet leafhoppers. Maximum total fecundity (*R*_*m*_) was estimated at 30.6°C, and beet leafhoppers reared on *K*. *scoparia* showed the highest *R*_*m*_ value followed by those on *P*. *ovata*, *E*. *cicutarium*, and *S*. *tragus*. Effects of temperature and host plant species on oviposition were modeled and successfully validated under semi-field and greenhouse conditions. This information is critical with regard to characterization of the relative importance of host plant species for beet leafhopper population growth under fluctuating temperature conditions. Modeling approaches presented here will also be valuable for the development of spatio-temporal decision support tools for beet leafhoppers in non-agricultural areas.

## Supporting information

S1 Table Summary of fluctuating temperature conditions for model validation. (DOCX)

S2 Table Statistical outputs of oviposition model validations for non-agricultural host plant species. (DOCX)
