# Supplementary material for: Modeling and validation of oviposition by a polyphagous insect pest as a function of temperature and host plant species
Source: PLoS One. 2022 Sep 2;17(9):e0274003. doi: 10.1371/journal.pone.0274003 (PMC9439214; doi:10.1371/journal.pone.0274003)
Supplement: S1 Table — (DOCX) [file pone.0274003.s001.docx]

**Table S1**. **Summary of fluctuating temperature conditions for model validation.**

| **Host plant** | **Replicate** | **Mean temperature ± SE (°C)** | **Minimum temperature (°C)** | **Maximum temperature (°C)** |
| --- | --- | --- | --- | --- |
| *Erodium cicutarium* | 1 | 14.12 ± 0.13 | 2.24 | 31.71 |
|  | 2 | 14.59 ± 0.14 | 3.69 | 31.71 |
| *Kochia scoparia* | 1 | 24.13 ± 0.20 | 19.97 | 29.27 |
|  | 2 | 24.06 ± 0.21 | 19.97 | 29.27 |
| *Plantago ovata* | 1 | 14.12 ± 0.13 | 2.24 | 31.71 |
|  | 2 | 14.05 ± 0.13 | 2.24 | 31.71 |
| *Salsola tragus* | 1 | 14.12 ± 0.13 | 2.24 | 31.71 |
|  | 2 | 14.05 ± 0.13 | 2.24 | 31.71 |
