# Supplementary material for: Modeling and validation of oviposition by a polyphagous insect pest as a function of temperature and host plant species
Source: PLoS One. 2022 Sep 2;17(9):e0274003. doi: 10.1371/journal.pone.0274003 (PMC9439214; doi:10.1371/journal.pone.0274003)
Supplement: S2 Table — (DOCX) [file pone.0274003.s002.docx]

**Table S2**. **Statistical outputs of oviposition model validations for non-agricultural host plant species.**

| **Host plant** | **Replicate** | **D.F.** | ***F* value** | ***P* value** |
| --- | --- | --- | --- | --- |
| *Erodium cicutarium* | 1 | 1, 9 | 27.10 | < 0.001 |
|  | 2 | 1, 9 | 6.86 | 0.028 |
| *Kochia scoparia* | 1 | 1, 14 | 40.08 | < 0.001 |
|  | 2 | 1, 11 | 20.15 | < 0.001 |
| *Plantago ovata* | 1 | 1, 10 | 21.79 | < 0.001 |
|  | 2 | 1, 10 | 35.97 | < 0.001 |
| *Salsola tragus* | 1 | 1, 9 | 11.72 | 0.008 |
|  | 2 | 1, 9 | 31.06 | < 0.001 |
